# Dynamic activity patterns in the anterior temporal lobe represents object semantics

**DOI:** 10.1080/17588928.2020.1742678

**Published:** 2020-04-06

**Authors:** Alex Clarke

**Affiliations:** Department of Psychology, University of Cambridge, Cambridge, UK

**Keywords:** ATL, RSA, semantics, perirhinal, time course

## Abstract

The anterior temporal lobe (ATL) is considered a crucial area for the representation of transmodal concepts. Recent evidence suggests that specific regions within the ATL support the representation of individual object concepts, as shown by studies combining multivariate analysis methods and explicit measures of semantic knowledge. This research looks to further our understanding by probing conceptual representations at a spatially and temporally resolved neural scale. Representational similarity analysis was applied to human intracranial recordings from anatomically defined lateral to medial ATL sub-regions. Neural similarity patterns were tested against semantic similarity measures, where semantic similarity was defined by a hybrid corpus-based and feature-based approach. Analyses show that the perirhinal cortex, in the medial ATL, significantly related to semantic effects around 200 to 400 ms, and were greater than more lateral ATL regions. Further, semantic effects were present in low frequency (theta and alpha) oscillatory phase signals. These results provide converging support that more medial regions of the ATL support the representation of basic-level visual object concepts within the first 400 ms, and provide a bridge between prior fMRI and MEG work by offering detailed evidence for the presence of conceptual representations within the ATL.

## Introduction

The Anterior temporal lobes (ATL) are considered a critical region in many theories of semantic memory, and function in a transmodal fashion (Bruffaerts et al., 2019; L. Chen et al., 2017; Clarke & Tyler, 2015; Damasio et al., 2004; Miyashita, 2019; Patterson et al., 2007; Lambon Ralph et al., 2017; Simmons & Barsalou, 2003; Taylor et al., 2011). This has been realized through important converging evidence across neuropsychology, functional brain imaging, computational modeling, brain stimulation and invasive neural recordings, each providing an overlapping perspective on the neural representation of semantic knowledge. The ATL has long been considered a multi-modal convergence zone (Damasio, 1989), processing conjunctive representations of increasing complexity compared to regions it receives input from (Cowell et al., 2019; Meyer & Damasio, 2009; Miyashita, 2019; Simmons & Barsalou, 2003). Whilst the ATL is known to be anatomically connected to different modality-specific pathways (Bajada et al., 2019; Guo et al., 2013; Papinutto et al., 2016; Simmons et al., 2010), allowing different routes between sensation and meaning representations, perhaps the most well-studied route is the access of semantic representations from visual objects. In this study, the aim is to test for converging evidence of the representational role of the ATL for visual object semantics, the timing when semantic representations are evoked, and the variation of semantic effects across lateral to medial ATL sub-regions.

Numerous studies have now demonstrated that semantically related items have similar patterns of activation within the ATL (Bruffaerts et al., 2013; Y. Chen et al., 2016; Clarke et al., 2018; Clarke & Tyler, 2014; Coutanche & Thompson-Schill, 2015; Kivisaari et al., 2019; Malone et al., 2016; Martin et al., 2018; Meyer & Damasio, 2009; Murphy et al., 2017; Peelen & Caramazza, 2012). Whilst these studies have used a variety of methods for determining semantic relatedness – ranging from superordinate category clustering to similarity between basic-level concepts – a powerful approach is to characterize the semantic similarity between individual concepts and compare this to the similarity of brain activations. For example, using the representational similarity analysis (RSA) framework, Clarke and Tyler (2014) showed that fMRI-activation pattern similarity in the perirhinal cortex and surrounding tissue was statistically related to the semantic similarity between objects. In this case, and in others, semantic similarity was defined based on the amount of overlapping semantic features associated with the concepts (e.g., *flies, is fast, has wings, made of metal* are features for the concept Airplane). The observed statistical correspondence between the semantic-feature similarity space and brain activation patterns was seen as evidence the perirhinal cortex represents semantic information about objects, with the dimensions of this cognitive space being well-modeled by feature dimensions. Such a relationship between a semantic-feature space and medial regions of the ATL are also seen for written words (Bruffaerts et al., 2013; Martin et al., 2018) and imagined concepts (Kivisaari et al., 2019), highlighting that such semantic representations are not explained by the physical visual stimulus similarity alone.

Research based on time-sensitive techniques has further pointed toward the access of basic-level conceptual knowledge for visual objects after around 200 to 400 ms (Bankson et al., 2018; Chan et al., 2011; Y. Chen et al., 2016; Clarke et al., 2015, 2018; Kreiman et al., 2000; Leonardelli et al., 2019; Mollo et al., 2017; Rogers et al., 2019; Rupp et al., 2017; Schendan & Ganis, 2012; Schendan & Maher, 2009; Sudre et al., 2012). The utilization of RSA with EEG, MEG and human intracranial recordings is becoming increasingly popular, highlighting the relationship between dynamic neural activity with low to high-level visual properties (Carlson et al., 2013; Cichy et al., 2016, 2014; Contini et al., 2017; Kaneshiro et al., 2015; Seeliger et al., 2018) and basic-level conceptual representations (Bankson et al., 2018; Y. Chen et al., 2016; Clarke et al., 2018). In particular, recent evidence suggests theta activity in the ATL might be particularly important for coding semantic representations of basic-level objects (Clarke et al., 2018), which is consistent with evidence that theta activity in the MTL dissociates between different object categories (Kraskov et al., 2007) and tracks the access of semantic knowledge (Ackeren et al., 2014; Bastiaansen et al., 2005; Fuentemilla et al., 2014; Halgren et al., 2015; Solomon et al., 2019; Watrous & Ekstrom, 2014).

Previous research points to a role for the ATL in supporting the dynamic construction of semantic representations over time, possibly through theta activity. However, in many of these cases, either (A) the spatial specificity afforded by fMRI has been lacking, resulting in inferences at the level of the ATL, or (B) the evidence is not situated at the level of individual concepts, but rather super-ordinate categories. The current study looks to overcome this, by probing the semantic nature of neural representations in the ATL at a spatially and temporally resolved scale, using a methodological and cognitive framework consistent with previous studies examining semantic-feature based representations of individual concepts through fMRI (Clarke & Tyler, 2014) and MEG (Clarke et al., 2018). Utilizing human intracranial recordings allows for the testing of semantic representations during object recognition in high resolution neural activation patterns from anatomically distinct sub-regions within the ATL, providing important converging evidence.

## Methods

The data used in this research was originally published by Morton et al. (2013) and is freely available from the Computational Memory Lab (http://memory.psych.upenn.edu/Electrophysiological_Data). Only the essential details for the participants, experimental paradigm and data collection are reproduced here, along with the specific methodological details relating to this study.

### Participants

Eleven patients with medication-resistant epilepsy underwent invasive ECoG and depth electrode monitoring for the clinical determination of the location of epileptogenic foci for subsequent resection. The research protocol was approved by the relevant institutional review boards, and informed consent was obtained from all participants.

For this study, eight of the eleven patients were analyzed as they had electrode contacts in all ATL sub-regions, with the exception of one patient who did not have contacts in the temporal pole (Table 1). One patient underwent invasive monitoring on two occasions with overlapping sets of electrodes and different trials, and consistent with Morton et al. (2013), the two sessions are treated as two separate data sets (ID 3 & 4).

**Table 1.** Patient and session details.

					Electrodes in ROIs				
ID	Age	Gender	Sessions	Items in property norms	Temporal pole	Middle temporal	Inferior temporal	Fusiform	Perirhinal
1	40	F	10	47	2	9	8	1	1
2	39	M	4	36	3	8	9	2	3
3	34	F	2	18	2	11	8	3	3
4	34	F	8	49	2	7	8	6	6
5	44	M	4	34	5	18	4	4	1
6	43	M	5	43	7	10	9	2	4
7	18	M	6	41	0	17	12	11	7
8	39	M	2	15	2	4	7	1	2

Details of each patient and the recording sessions, along with number of stimuli in the property norms and electrodes in each ROI

### Materials and experimental paradigm

Stimuli were color and grayscale photographs of famous landmarks, celebrity faces, and common objects, with the name of the stimulus presented in text above the picture. In this study, only the objects are analyzed with the landmarks and faces treated as filler trials. All objects reflected common non-living objects/artifacts.

Participants were presented with lists of 9 items, with 3 items of each type (objects, faces, landmarks) presented in a pseudorandom order. Before each item, a text cue indicating the type (e.g., face) of the upcoming item was shown for 1000 ms, and a 200–500 ms ISI before presentation of the item for 3500 ms. During stimulus presentation, participants made a 4-point semantic judgment (for objects: ‘How often do you come across this object in your daily life?’). Each stimulus was followed by a blank ISI of 1000 ± 200 ms.

After presentation of the last stimulus, the screen was blank for 1300 ± 100 ms, followed by presentation of a row of asterisks and a 300-ms tone signaling the start of a 60 s immediate free recall period. Participants were presented with 20 lists in each session (session totals: 60 object images, 12 unique concepts), and each participant completed 1–10 sessions (see Table 1 for the number of sessions completed by each participant). Five different images of the same item were shown within a session.

The object images were of 72 different concepts, with 5 images used for each concept. Of these, 51 were also found in a published set of concept property norms (Devereux et al., 2014) allowing for the extraction of semantic feature information for these items. As different participants completed a different number of sessions, the number of concepts/items analyzed varied across participants (Table 1). All data analyses are restricted to these items.

### ECoG recording and data processing

ECoG was recorded using a Grass Telefactor or Nicolet digital video-EEG system, and sampled at 400 Hz (in one case at 512 Hz). Data preprocessing used Fieldtrip and EEGlab functions. The data were notch filtered to remove line noise at 60 Hz and the harmonics (120 and 180 Hz), before epoching between −1.5 and 3 seconds and baseline corrected using the mean pre-stimulus response between −500 and 0 ms. Bad channels were identified by visual inspection, and removed from the data (mean 3.5% of channels), and a common average reference was applied.

Independent components analysis was used for the removal of components associated with saccades (Kovach et al., 2011), implemented with runica, extracting N components, where N was 75% of the total number of good electrodes. To focus the IC activations on the frequency range associated with saccades in ECoG channels, the activations were filtered for gamma-band activity between 20 and 190 Hz, before convolving the filtered activations with a saccade-related potential template and calculating the number of saccade events per second (Craddock et al., 2016). The two components with the highest number of saccade events were discarded, and the data reconstructed based on the remaining non-filtered component activations. The resulting data were averaged over the five repetitions of each object, combined across sessions and restricted to those items that were in the property norms.

### Electrode localization and selection

**Figure 1.**
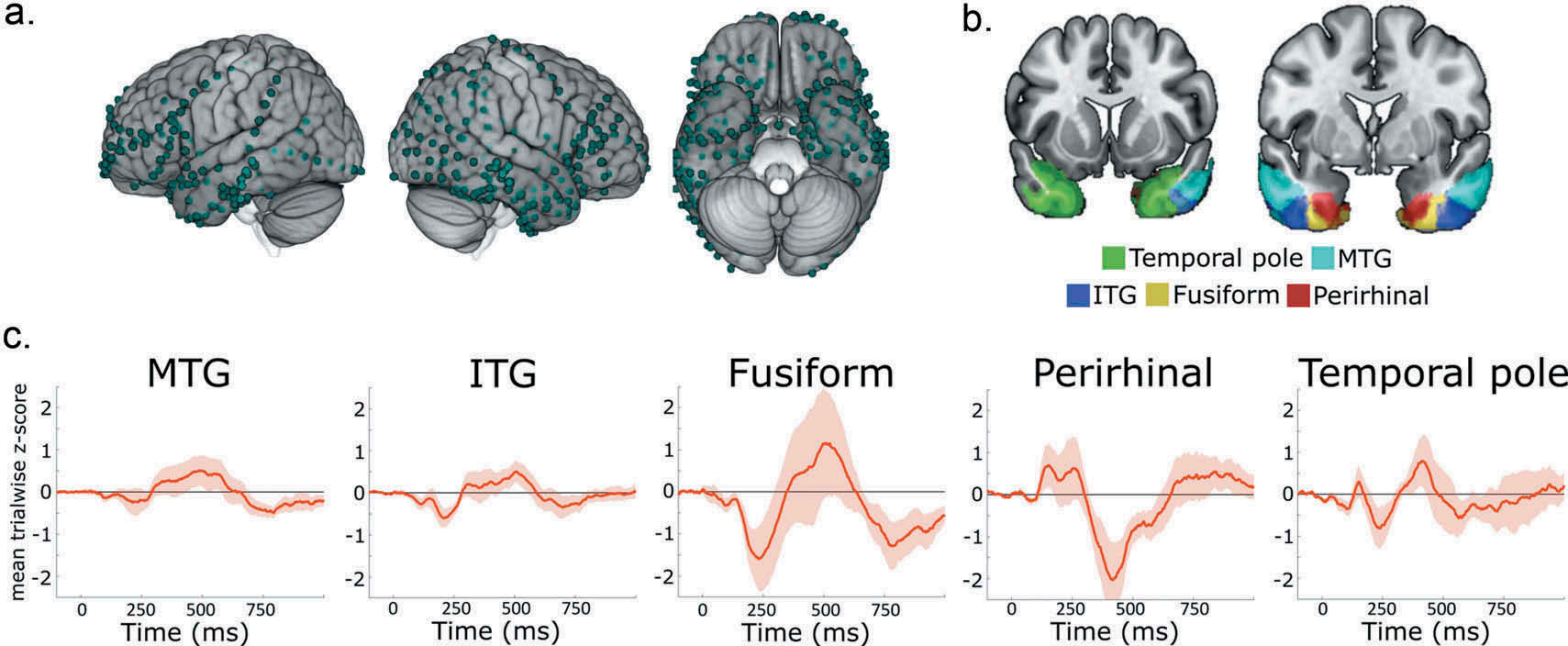
Anterior temporal lobe regions. A) Electrode contacts across the brain for the 8 patients. B) ROIs in the ATL (MNI y = 9 and y = 1). C) Evoked activity in each ROI. Plots show the group mean response from the z-scored single trial activity. Shaded areas show ±1 standard error of the mean.

Electrode grids and depth electrodes were positioned based on the decisions of the clinical team and not for research specific purposes (Figure 1(a)). To localize the positions of the electrodes, an indirect stereotactic technique was used based on co-registered post-operative computed tomography and pre- or post-operative magnetic resonance imaging, and converted locations into MNI coordinates (Morton et al., 2013).

Here, five ATL ROIs were used from the anterior temporal lobe atlas created by Wright et al. (2015) – the middle temporal gyrus (MTG), the inferior temporal gyrus (ITG), the temporal pole (TP), the anterior fusiform (Fus) and the perirhinal cortex (PRC; Figure 1(b)). Briefly, ROIs were manually traced on 15 normalized high resolution structural images and combined to create a probability atlas. Each voxel was assigned to the region with the highest probability. Using these ROIs, electrodes were selected within the anterior temporal lobes bilaterally.

### Representational similarity analysis

#### Calculating semantic similarity

RSA was used to compare the semantic similarity between items with the similarity based on the ECoG signals. Semantic-feature similarity was based on combining data from a published set of property norms (Devereux et al., 2014) and word2vec – a distributional corpus-based model of word meanings. Although semantic feature spaces can be defined based solely on either property norms or corpus-based statistics, recent fMRI evidence suggests combining the approaches can be an effective way of modeling multidimensional conceptual spaces (Kivisaari et al., 2019) (see Supplementary Figure S1 for a comparison between approaches).

**Figure 2.**
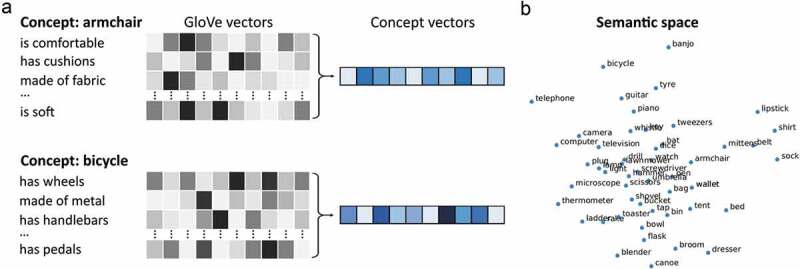
**Semantic similarity space**. A) Concept vectors were created from the average of GloVe vectors for the semantic-features of the concept. B) MDS of the resulting semantic similarity space.

The property norms were a version of the Center for Speech, Language and the Brain norms (available from https://cslb.psychol.cam.ac.uk/propnorms) which specify how 826 different concepts relate to 3026 different features (e.g., *is comfortable, has cushions, is sat on* are features of an armchair). Using these property norms, each concept can be represented by a list of features that collectively define the concept. For each feature (e.g., *comfortable, cushions, sat* from the above example), a vector of length 300 was obtained using the pre-trained word2vec model GloVe (Pennington et al., 2014) (Figure 2(a)). Each vector is derived from word-word co-occurrences from large text corpora, with the result that words which co-occur frequently in the same language context will have similar vector representations. A semantic vector for a concept was then calculated as the average of the feature vectors, resulting in a 300 dimension vector reflecting the combined semantic-feature information for that concept. Finally, the semantic similarity space for all concepts was calculated as the cosine distance between all possible pairs of concepts (Figure 2(b)).

#### Evaluating semantic similarity in the brain

The similarity of the ECoG signals was calculated for electrodes within each ROI separately. Within each ROI, single item activity was selected for each electrode within the ROI. RDMs between items were calculated using cosine distance for every time-point between −100 and 1000 ms based on a spatiotemporal pattern defined by the number of electrodes in the ROI and data from ±50 ms either side of the current time point (Y. Chen et al., 2016; Tyler et al., 2013a). A further analysis was conducted which targeted a time window between 200 and 400 ms, which is highly associated with accessing conceptual knowledge for object concepts (Bankson et al., 2018; Chan et al., 2011; Y. Chen et al., 2016; Clarke et al., 2015, 2018; Kreiman et al., 2000; Leonardelli et al., 2019; Mollo et al., 2017; Rogers et al., 2019; Rupp et al., 2017; Schendan & Ganis, 2012; Schendan & Maher, 2009; Sudre et al., 2012).

For the similarity based on time-frequency representations (TFRs), oscillatory phase was calculated for each item and for every electrode within the ROI using Morlet Wavelets (timefreq.m function in EEGLAB). Oscillatory phase signals were extracted between 200 and 400 ms in 20 ms time steps, and between 4 and 190 Hz in 60 logarhythmically spaced frequency steps. A 5-cycle wavelet was used at the lowest frequency, increasing to a 15 cycle wavelet at the highest. This produced a TFR for every item at every electrode in the ROI. RDMs between item TFRs were calculated as the circular distance between items (Berens, 2009) at each time/frequency point.

For each participant, the RDMs based on ECoG signals were tested against the semantic RDM using Kendall’s Tau-A. Random effects analysis testing for positive RSA effects was conducted for each time point using a Wilcoxon test against zero (alpha 0.05). Cluster-mass permutation testing was used to assign p-values to clusters of significant tests (Maris & Oostenveld, 2007). For each permutation, the sign of the RSA correlations was randomly flipped for each participant before Wilcoxon tests of the permuted data at each data point. The cluster p-value for clusters in the original data were defined as the proportion of the 10,000 permutations (plus the observed cluster-mass) that was greater than or equal to the observed cluster-mass. When testing for effects within the 200–400 ms time window, RSA effects were first averaged over time before using a Wilcoxon test against zero. When comparing between RSA effects across regions, a two-sample Wilcoxon test was used (alpha 0.025). For the analysis of oscillatory phase, RSA effects were averaged within the time window of interest (200 to 400 ms) and for each frequency band (theta: 4–8 Hz, alpha: 9–14 Hz, beta: 15–30 Hz, low gamma: 30–70 Hz, and high gamma: 70–150 Hz).

## Results

The primary question in this study was to test for the presence of semantic object representations across the lateral to medial aspect of the anterior temporal lobes. Semantic relations between objects were calculated using a feature-based approach to defining semantic information associated with each object, that was combined with a distributional word model of semantics (Figure 2). The semantic similarity across object concepts was then compared with activity pattern similarity from each anatomically defined ROI.

**Figure 3.**
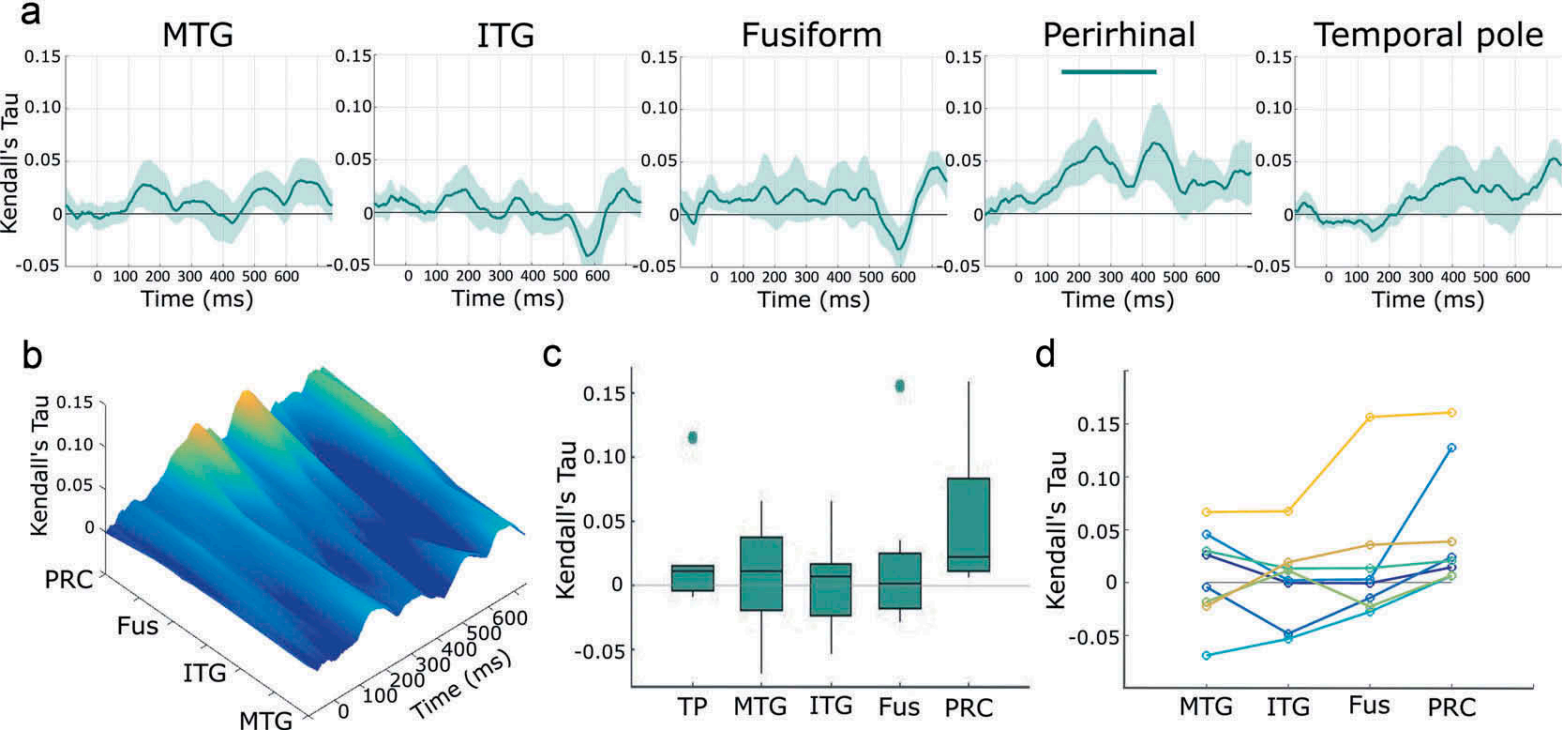
**Semantic similarity effects across the anterior temporal lobe**. A) Shaded areas show ±1 standard error of the mean, horizontal bar shows statistically significant cluster. B) RSA effect across the lateral to medial axis of the ATL. C) RSA effects within the 200–400 ms time window displayed as boxplots. Horizontal lines show the median, with box edges showing the 25^th^ and 75^th^ percentiles. Outliers shown as separate points. D) Individual patient RSA effects across the ROIs on the lateral to medial ATL axis. Also see Supplementary Figure 1.

The first analysis tested for the presence of semantic similarity effects in each ATL region across time. Using RSA, significant semantic-feature similarity effects were observed in the perirhinal cortex peaking near 250 and 450 ms (significant time window: 141–448 ms; cluster p = 0.007; Figure 3(a)). No significant semantic-feature similarity effects were observed in any other anterior temporal lobe region, and peaks were prominent for the medial perirhinal in contrast to more lateral regions of the anterior temporal lobe (Figure 3(b)).

To address the question of whether PRC semantic effects were larger than in other ATL regions, RSA effects were extracted from each region between 200 and 400 ms. This particular time window was chosen due to prior research implicating this time frame (Bankson et al., 2018; Chan et al., 2011; Y. Chen et al., 2016; Clarke et al., 2015, 2018; Kreiman et al., 2000; Leonardelli et al., 2019; Mollo et al., 2017; Rogers et al., 2019; Rupp et al., 2017; Schendan & Ganis, 2012; Schendan & Maher, 2009; Sudre et al., 2012) and to avoid circular inference. PRC semantic effects were significantly greater than all ATL sub-regions except the temporal pole (Figure 3C); fusiform (p = 0.0078, PRC greater in 8/8 participants), inferior temporal (p = 0.016, PRC greater in 7/8 participants), middle temporal (p = 0.0391, PRC greater in 6/8 participants) but not the temporal pole (p = 0.078, 6/7 participants). As this shows, the increased PRC effects were consistently seen across participants (Figure 3D).

**Figure 4.**
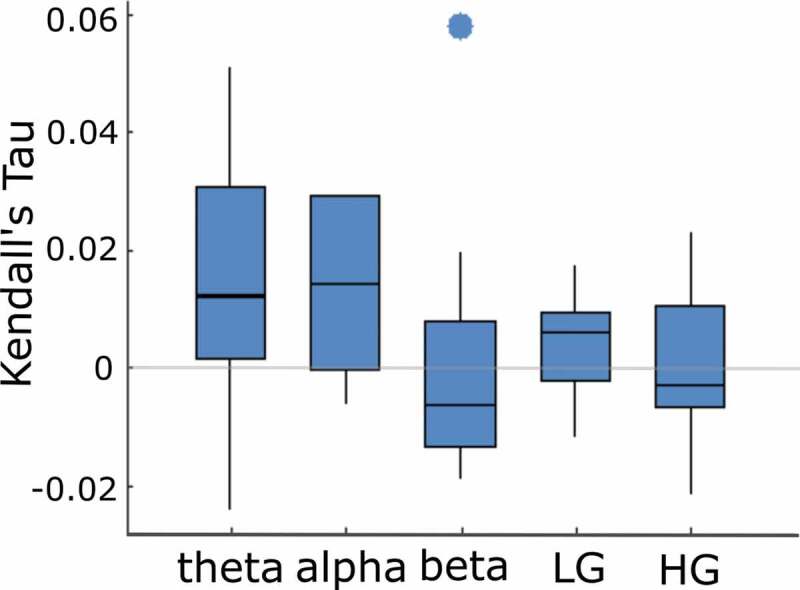
**Semantic similarity effects across frequency bands in the perirhinal cortex**. RSA effects within the 200–400 ms time window displayed as boxplots. Horizontal lines show the median, with box edges showing the 25^th^ and 75^th^ percentiles. Outliers shown as separate points.

Finally, to probe the nature of perirhinal semantic effects further, RSA effects were calculated from oscillatory phase signals in different frequency bands within our pre-defined time window (200–400 ms). Significant semantic effects were present in both theta (4–8 Hz; p = 0.0391, positive effects in 7/8 participants) and alpha activity (8–14 Hz; p = 0.0391, positive effects in 7/8 participants), but not in beta (15–30 Hz; p = 0.68), low gamma (30–70 Hz; p = 0.23) or high gamma signals (70–150 Hz; p = 0.53) (Figure 4). This suggests that low frequency phase activity patterns might underlie semantic similarity effects in the perirhinal cortex, and is consistent with previous MEG evidence using a highly similar approach that indicated ATL theta phase patterns were associated with semantic similarity effects (Clarke et al., 2018).

## Discussion

The anterior temporal lobes play a prominent role in many theories of semantic cognition, with different accounts placing a different emphasis on the contribution of individual cortical regions, or graded contributions across the lobe (Barense et al., 2011; Clarke & Tyler, 2015; Damasio et al., 2004; Grabowski et al., 2001; Mehta et al., 2016; Patterson et al., 2007; Lambon Ralph et al., 2017). Despite this, the contribution of exact sub-regions has not always been clear (Bonner & Price, 2013). This research examined the representation of object semantics within sub-regions of the anterior temporal lobes. Using representational similarity analysis and quantifying semantic similarity, it was shown that the perirhinal cortex represented object-specific semantics beyond around 150 ms, which was not observed in any other ATL subregion. This suggest that the perirhinal region is the most important sub-region of the ATL in representing the semantics of individual visual objects.

This research looked to build-upon previous studies relating semantic-feature similarity effects to neural similarity, that suggested the perirhinal cortex uniquely represented semantic item information (Bruffaerts et al., 2013; Clarke & Tyler, 2014; Kivisaari et al., 2019; Martin et al., 2018), but perhaps in cohort with the temporal pole (Martin et al., 2018). Further, EEG and MEG studies point to semantic-feature effects for individual items beyond around 200 ms (Bankson et al., 2018; Clarke et al., 2015, 2018; Leonardelli et al., 2019; Mollo et al., 2017; Schendan & Maher, 2009; Sudre et al., 2012) but lacked detailed spatial specificity. It should be stressed that these studies, along with the current study, probe the semantics of objects at a basic-level (e.g., basketball, lawnmower) rather than a superordinate category level (e.g., tool), which may be associated with more posterior regions of the VVP (Bi et al., 2016; Connolly et al., 2012; Devereux et al., 2018; Konkle & Caramazza, 2013; Peelen & Downing, 2017; Tyler et al., 2013b) at earlier points in time (Clarke et al., 2015; Mace et al., 2009).

**Figure 5.**
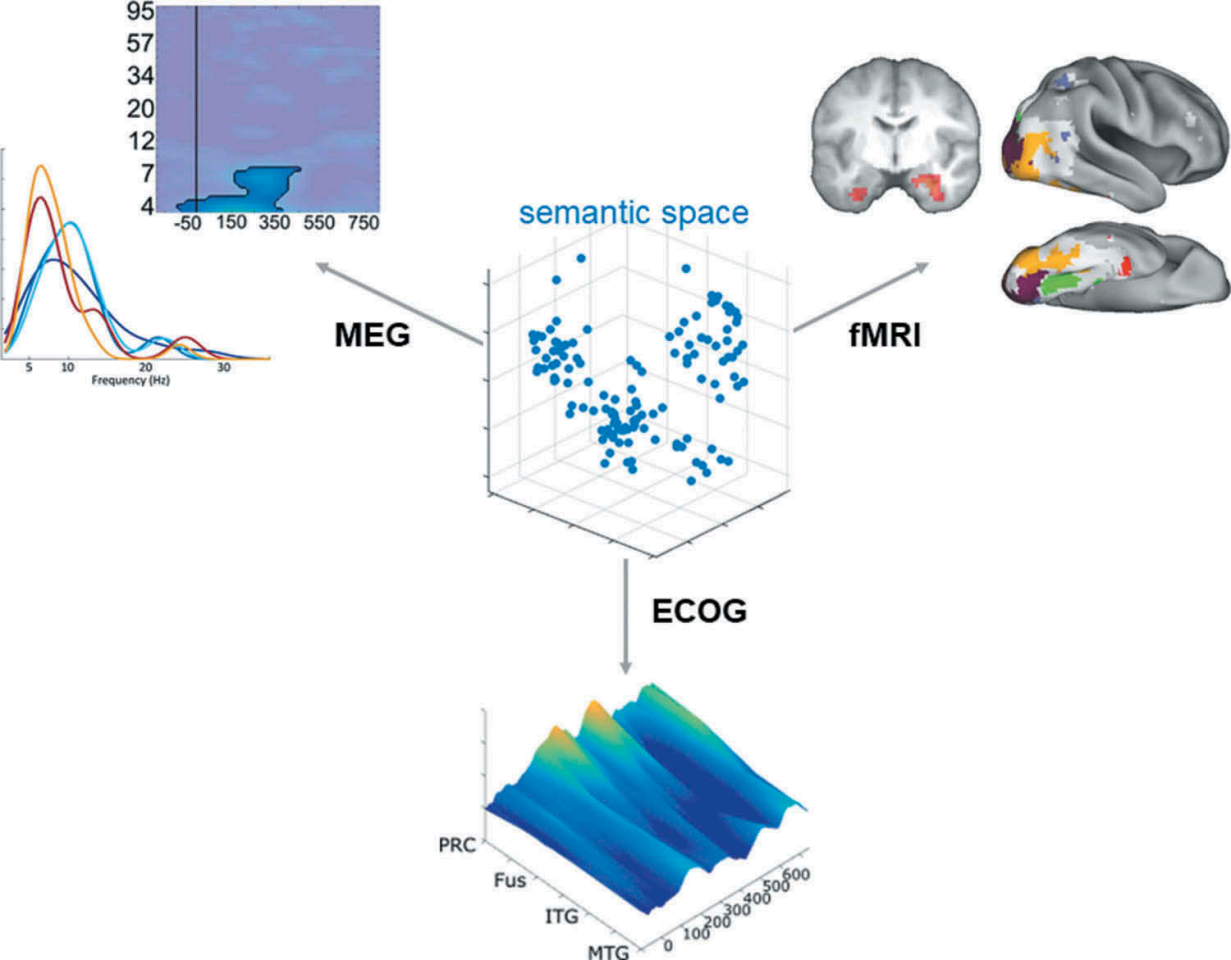
**Converging evidence points to semantic-feature similarity effects in the perirhinal cortex around 150 to 450 ms**. MEG evidence shows semantic-feature RSA effects peaking in theta frequencies (yellow/red curves) compared to visual effects peaking near alpha (blue curves). Time-frequency plot shows RSA effects of semantic-features in the left ATL region. fMRI evidence shows semantic-feature effects in bilateral medial anterior temporal regions overlapping with the perirhinal cortex. Inflated brain map shows semantic-feature effects (red) in relation to superordinate category effects (yellow/green) and a model of V1/V2 (purple). fMRI data reproduced from (Clarke & Tyler, 2014), MEG data reproduced with permission, Alex Clarke, 'Oscillatory dynamics of perceptual to conceptual transformations in the ventral visual pathway', Journal of Cognitive Neuroscience, 30:11 (2018), pp. 1590-1605.  © 2018 by the Massachusetts Institute of Technology.

This research study moved beyond previous work by testing for temporally and spatially specific semantic information using intracranial recordings from human anterior temporal lobe sub-regions. This study suggests that the perirhinal cortex in the medial aspect of the anterior temporal lobe is likely to be the most important in generating semantic representations for visual objects between around 200 and 400 ms. Further, such semantic effects were significantly stronger than more lateral ATL regions. Overall, the converging nature of the evidence presented is significant, providing a level of spatial and temporal specificity that our previous fMRI (Clarke & Tyler, 2014) and MEG (Clarke et al., 2018) results could not provide alone (Figure 5).

The results of this study show both similarities and divergence from a previously reported study of semantic similarity effects in anterior temporal lobe activations. Chen et al. (2016) used RSA with intracranial recordings to show that activity in the ventral anterior temporal lobe reflected the semantic similarity of objects, where semantic similarity was quantified based on semantic-feature similarity. Here, semantic similarity is also reflected in anterior temporal lobe activity, but with a focus on separating the contributions of specific cortical sub-regions, based on the anatomy. This suggests that while ventral aspects of the anterior temporal lobe are crucial for object semantics, the focus of the effects here is situated in more medial perirhinal cortex. The current study is also consistent with a graded profile of semantic effects across the ATL, which could support a semantic gradient along the ventral-medial axis in the anterior temporal lobe, consistent with previous suggestions (Rice et al., 2015; Visser et al., 2012). This may also extend posteriorly, where semantic similarity effects appear to transition to be focussed on the fusiform gyrus as we shift away from the anterior temporal lobe.

The finding that semantic-feature similarity is reflected in low frequency phase patterns echoes recent MEG evidence of semantic similarity in ATL theta phase patterns (Clarke et al., 2018). The converging evidence for the relevance of theta phase coding in medial ATL, using the same analytical approach, supports the conclusion that low frequency (in particular, theta) activity patterns in perirhinal cortex represents object semantics. The relationship between theta activity in ventral and perirhinal regions and semantics has further been reported by Halgren et al., (2015) who suggested that theta activity in the ventral ATL and peri/entorhinal cortex largely reflects an alternation of feedforward inputs and top-down effects in aid of memory formation. In this manner, theta allows the widespread integration across cortical networks (Halgren et al., 2015).

The timing of the perirhinal semantic effects is similar to reported single unit activity in the medial temporal lobe that is highly sensitive to individual concepts, regardless of the nature of the input (Quian Quiroga et al., 2009; Quiroga et al., 2005). Such concept cells are considered to be part of a sparse and distributed code where many cells respond to a specific concept, and related concepts will active a partially overlapping population of cells (Quian Quiroga, 2016, 2012), giving rise to a distributed spatial pattern where the degree of overlapping cells could relate to conceptual overlap. However, these MTL concept responses are not likely to be driven by an initial feedforward input from the visual pathway (as the latency is closer to 300 ms), and are claimed to be important for the formation of memories concerning concepts, rather than a semantic representation of an item required for recognition (Quian Quiroga, 2012). Consistent with this, perirhinal semantic effects, as seen here, could relate to accessing the object semantics from perceptual inputs through recurrent activity, and further act to support the episodic encoding of the item in coordination with the hippocampus and other MTL regions (Backus et al., 2016; Fell et al., 2001; Halgren et al., 2015; Miller et al., 2018; Quian Quiroga, 2012; Staresina et al., 2012; Staresina & Wimber, 2019; Watrous & Ekstrom, 2014), allowing for the binding of the item’s semantic properties with the ongoing spatio-temporal context (Ekstrom & Ranganath, 2018; Ranganath & Ritchey, 2012; Staresina & Wimber, 2019; Watrous & Ekstrom, 2014).

The observed effects reported here are despite the small number of subjects involved (n = 8 in all but one sub-region), the variable number of electrodes in the regions (1–11 per sub-region), and the small number of concepts available (see Table 1). In addition, all the concepts here are non-living artifacts, while it is often considered that the perirhinal is most critical for the recognition of living things (Taylor et al., 2011). Each of these issues could lead to a weakening of the semantic similarity effects observed, and the effects could be even stronger if these aspects improved. In addition, the visual properties of the concepts were not examined in this study as the neural patterns were based on an average over 5 different images of each concept. However, future works could further establish the oscillatory relationship between visual and semantic properties across ATL sub-regions.

In conclusion, this study provides important converging evidence for the role of the perirhinal cortex in representing the semantics of basic-level visual object concepts. Together with previous studies using the same statistical framework and approach, it is suggested that the perirhinal cortex is the most important region in the ATL for representing the semantics of visual objects, with these representations being activated beyond around 150 ms.
